# Overexpression of transketolase-like gene 1 is associated with cell proliferation in uterine cervix cancer

**DOI:** 10.1186/1756-9966-28-43

**Published:** 2009-03-30

**Authors:** Hui Chen, Jian-Xin Yue, Shou-Hua Yang, Hui Ding, Rong-Wei Zhao, Song Zhang

**Affiliations:** 1 Department of gynecology, Union Hospital of Tongji Medical College, Huazhong University of Science & Technology, 1277 Jiefang Dadao Avenue, Wuhan, Hubei, PR China; 2 Department of Otolaryngology, Union Hospital of Tongji Medical College, Huazhong University of Science & Technology, 1277 Jiefang Dadao Avenue, Wuhan, Hubei, PR China

## Abstract

**Background:**

Tumor cells need large energy and nucleic acids to proliferate and grow. For most of their energy needs, cancer cells depend more on glycolysis. For most of their nucleic acids needs, cancer cells depend more on the nonoxidative pathway of the pentose phosphate pathway. Transketolase(TKT) is a crucial enzyme in the nonoxidative pathway of the PPP.

**Methods:**

The real-time quantity PCR was used to determine the expression of transketolase gene family in uterine cervix cancer. Transketolase activity of cell was determined by using enzyme-linked method. Cell proliferation was detected by using MTT.

**Results:**

The TKTL1 mRNA was specifically over-expressed in uterine cervix cancer cells(HeLa cell line) compare with normal human endocervical epithelial cells(End1/E6E7 cell line)(P < 0.05), whereas the expression of TKT and transketolase-like gene 2(TKTL2) have no significant differences between the two cell lines(P > 0.05). Moreover, we found that total transketolase activity was significantly reduced, and cell proliferation was remarkably inhibited after anti-TKTL1 siRNA treatment in HeLa cells. The total transketolase activity and cell proliferation have no significant differences after anti-TKTL1 siRNA treatment in End1/E6E7 cells.

**Conclusion:**

These results indicate that TKTL1 plays an important role in total transketolase activity and cells proliferation in uterine cervix cancer.

## Background

Tumor cells need more energy than normal cells to survive and grow. For most of their energy needs, normal cells rely on a process called respiration, which consumes oxygen and glucose to make energy-storing molecules of adenosine triphosphate (ATP). But cancer cells typically depend more on glycolysis, the anaerobic breakdown of glucose into ATP [1]. Warburg had identified a particular metabolic pathway in carcinomas characterized by the anaerobic degradation of glucose even in the presence of oxygen (known as the Warburg effect) 80 years ago [2]. Although the molecular basis for the altered glucose metabolism has not been identified yet, widespread clinical use of positron-emission tomography (PET) has confirmed that there exists enhanced glucose degradation in tumors [3]. At the annual meeting (2006) of American Association of Cancer Research, Gottlieb launched a lecture with this provocative claim: "I believe I'm working on the seventh element, which is bioenergetics."

Tumor cells need large energy and nucleic acids to proliferate and grow. The pentose phosphate pathway (PPP) is an important pathway in glucose metabolism. Transketolase is a crucial enzyme in the nonoxidative pathway of the PPP. It plays a crucial role in nucleic acid ribose synthesis utilizing glucose carbons in tumor cells. Boros[4] found that more than 85% of ribose recovered from nucleic acids of certain tumor cells is generated directly or indirectly from the nonoxidative pathway of the PPP. Three human transketolase genes have been recognized: they are transketolase(TKT), transketolase-like gene 1 (TKTL1) and transketolase-like gene 2 (TKTL2). Langbein[5] found that TKTL1 mRNA and protein are specifically over-expressed in tumors, whereas TKT and TKTL2 expression are not upregulated. Staiger[6] found that the upregulation of TKTL1 is a common phenomenon in gastric cancer and cancer of the gastroesophageal junction leading to an enhanced, oxygen-independent glucose usage which might contribute to a more aggressive tumor growth.

Uterine cervix cancer is a common tumor in women. Diffusion and metastasis play an important role in unfavourable prognosis of uterine cervix cancer. We knew little about the mechanism of invasion and metastasis in uterine cervix. Kohrenhagen[7] found that TKTL1 plays an important role in the progression of cervical neoplasia. But, the relative contributions of TKTL1 gene to energy metabolism and cell proliferation in uterine cervix cancer have not been investigated. In the present study, the relationship between transketolase-like gene 1 and transketolase activity or cell proliferation was investigated in uterine cervix cancer. These results indicate that TKTL1 gene influences cell proliferation by regulating total transketolase activity in human uterine cervix cancer cells.

## Materials and methods

### Reagent and Instrument

DMEM, Lipofectamine™ 2000 and Trizol were obtained from Invitrogen Co (Carlsbad, CA, USA); Keratinocyte serum-free medium (KER – SFM) were obtained from GIBCO (New York, USA). ReverTraAce-α-™ (Reverse transcription kit) were obtained from TOYOBO CO (Osaka, Japan); Quanti Tect™ SYBR Green PCR kit was purchased from Qiagen GmbH (Hilden, Germany); Coomassie Brilliant Blue G-250 was purchased from Amresco(USA);D-Ribose 5-phosphate disodium salt, xylulose 5-phosphate doium salt, triose-phosphate isomerase (TPI) and NADH were obtained from Sigma Co (St Louis, MO, USA); FAC-Scan Flow Cytometer (Becton Dickinson, USA); LightCycler Real-Time PCR Instrument (Roche, Switzerland); Olympus AU-2700 Autoanalyser (Toshiba, Japan).

### Cell culture

HeLa cell line was obtained from the American Type Culture Collection (ATCC). It was originally established from human cervix adenocarcinoma. Normal human endocervical epithelial cell line (Endl/E6E7) was obtained from Harvard Medical School. It was established by Fichorova from normal human endocervical epithelial tissue in 1997[8]. HeLa cells were cultured in DMEM, Endl/E6E7 cells were cultured in KER-SFM medium supplemented with 10%FCS at 37°C with 5% CO_2_.

### Plasmid construction

The candidate siRNA sequence specific for human TKTL1 mRNA was selected and designed by using online tools from Genesil Biotechnology Company. The selected candidate siRNA sequences were also checked to avoid any possible match with other genes or polymorphism of the target gene by Blast search. Three siRNA targeting human TKTL1 mRNA (National Center for Biotechnology Information access number: BC025382) and one scrambled siRNA (used for a negative control) with the following sequences were used: TKTL1 siRNA no.1, GCAGTCAGATCCAGAGAAT; TKTL1 siRNA no.2, GTTGGCATGCAAAGCCAAT; TKTL1 siRNA no.3 CAACAGAGTCGTTGTGCTG; negative TKTL1 siRNA control, GACTTCATAAGGCGCATGC. All siRNA sequences were synthesized by Wuhan Genesil Biotechnology Company, Wuhan, China. Synthetic sense and antisense oligonucleotides constitute the template for generating RNA composed of two identical 19-nt sequence motifs in an inverted orientation, separated by a 9-bp (TTCAAGACA) spacer to form a double strand hairpin of siRNA. Two micrograms of both oligonucleotide were annealed for 3 minutes at 94°C, for 30 minutes at 37°C, and for 10 minutes at 65°C, then ligated into 2 μg of pEGFP-C1-U6 plasmid (containing kanamycin resistance gene; the mouse U6 RNA Polymerase III promoter; enhanced green fluorescence protein clone) linearized with BamHI and HindIII. These constructs were cloned to competent *Escherichia coli*, according to the manufacture's instructions (Invitrogen). The sequences of the insert was confirmed by automated sequencing and by analyzing the fragments generated from digestion with BamHI. The resultant plasmids containing siRNA sequences 1, 2, 3 and negative control sequences were named pSih TKTL1-1, pSih TKTL1-2, pSih TKTL1-3 and pNC, respectively.

### Transfection

HeLa Cells and End1/E6E7 cells were stably transfected with three TKTL1 siRNA and a negative control siRNA in presence of Lipofectamine 2000 on 6-well plates according to the manufacturer's instruction, respectively. Transfected cells were selected for neomycin resistance in DMEM containing G418 for 4 weeks. Surviving colonies were isolated and expanded. These cells were harvested and TKTL1 mRNA levels were analyzed by real-time PCR at 96 h after cultured. Of the three plasmids tested, only one gave rise to over 80% inhibition of TKTL1. We select the plasmid named pSih TKTL1 to transfect HeLa Cells or End1/E6E7 cells in the posterior experiment. The negative control siRNA plasmid (without the shRNA coding DNA) did not show any significant level of TKTL1 reduction.

### RT-PCR

Total RNA was extracted from above-mentioned cells by using Trizol reagent according to the manufacturer's instructions. ReverTraAce-α-™ reverse transcription kit was used for reverse transcription following instruction manual. Real-time analysis was carried out on a Light Cycler Real-Time PCR Instrument by using SYBR Green I dye according to the manufacturer's protocol. Reactions were performed in a 25 μL volume. Real-time PCR was conducted by using the following parameters: denaturing at 94°C for 3 min, 40 cycles at 94°C for 5 s and at 57°C for 5 s. β-actin gene was used as an internal control and each assay included standard samples in duplicates. Data analysis was carried out by using LightCycler Data Analysis Software. In addition, PCR products were gel-separated to confirm the bands of the expected size. Basically, quantitative values are obtained from the threshold cycle number at which the increase in the signal associated with an exponential growth of PCR products starts to be detected. Final results, expressed as N-fold differences in target gene expression relative to the reference gene GAPDH, termed 'Ntarget', were determined as follows:

Ntarget = 2^(delta Ct sample - delta Ct reference gene)^.

Where delta Ct values of the sample and reference were determined by subtracting the average Ct value of the test gene from the average Ct value of the β-actin gene. The sequence of primer for three known human transketolase genes and β-actin were from reference.4. β-actin gene was amplified as internal control. The sequences of primers for TKT, TKTL1, TKTL2 were obtained by referring to Coy *et al *[9]. The sequences of primers for β-actin gene: 5'-GTG CGT GAC ATT AAG GAG-3'(sense), 5'-CTA AGT CAT AGT CCG CCT-3'(antisense) were designed by using Primer Premier 5.0 software package. The amplification conditions: denaturing at 94°C for 3 min, 40 cycles at 94°C for 5 s and at 57°C for 5 s. The amplification products were visualized by electrophoresis on a 1.5% agarose gel stained with ethidium bromide.

### Measurements of transketolase activity

In order to prepare the extract of HeLa and End1/E6E7 cells, cells were sonicated and centrifuged. The resulting supernatant was filtered to remove some endogenous metabolites. TK activity was determined by using enzyme-linked method [4]. Samples were added to a cuvette containing buffer (50 mM Tris/HCl, pH 7.6), 2 mM ribose 5-phosphate, 1 mM xylulose 5-phosphate, 5 mM MgCl_2_, 0.2 U mL^-1 ^of TPI, 0.2 mM NADH and 0.1 mM TPP. Reactions were initiated by the addition of HeLa or End1/E6E7 cells extract at 37°C. TK activity was expressed as ng product per min per mg total protein. Total protein content of cell extracts was determined by the Bradford method. Each experiment was repeated three times.

### Cell cycle analysis

10^4 ^cells of each group were seeded into a 6-well culture plate. Then cells were harvested after cultured for 72 hours. The harvested cells were washed with PBS, fixed with 70% alcohol, treated with RNase A and then stained with propidium iodide. The analysis of cell cycle distribution was performed by FAC-Scan Flow Cytometer (Becton Dickinson, USA) and analyzed by CellQuest software package. Each experiment was repeated three times.

### Cell proliferation assay

Cell proliferation was measured by the MTT assay. HeLa and End1/E6E7 cells (cells without transfection, cells transfected with control plasmid and cells transfected with siRNA), at 2 × 10^3 ^per well, were seeded into five 96-well culture plates, respectively. Each plate has three kinds of cells (without transfection, transfected with control plasmid or siRNA plasmid) and each group consisted of 12 parallel wells. Absorption value of one of five culture plates was determined by MTT at 490 nm after 24-hour cultivation. Then, absorption value of every culture plate was detected in the following four days. The growth curve of each group was plotted on the basis of absorption values. All experiments were done three times.

### Statistical analysis

All results are expressed as means ± SD. Statistical analysis was performed using the SPSS 10.0 software package for Windows, and statistical significance was set at *P *< 0.05.

## Results

### Effects of TKTL1 siRNA on the Expression of transketolase gene family members in the human uterine cervix cancer and normal cervical epithelial cells

**Figure 1:**
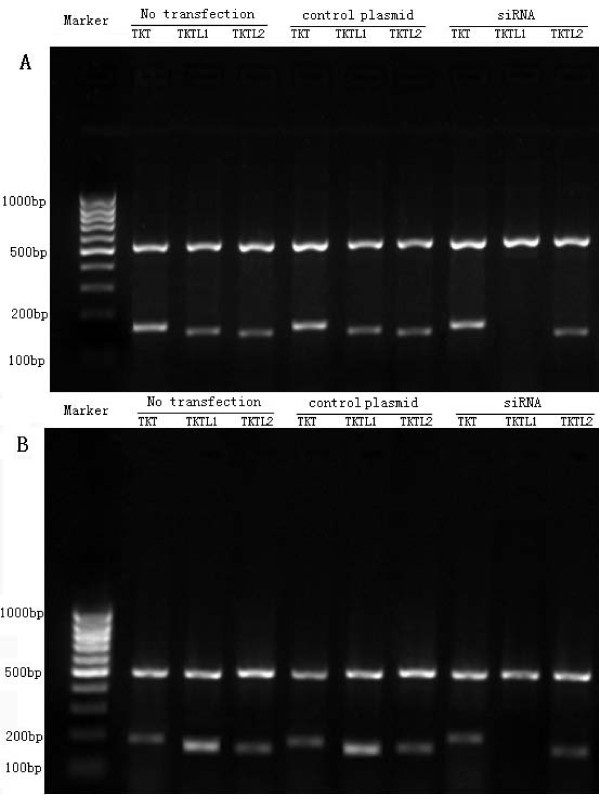
**Expression of transketolase gene family was analyzed by using gel electrophoresis in the End1/E6E7 cells and HeLa cells**. In the End1/E6E7 cells (A), the expression of TKT was significantly higher than the expression of TKTL1 and TKTL2. In the HeLa cells (B), the expression of TKTL1 was significantly higher than the expression of TKT and TKTL2. No expression of TKTL1 was found after transfected with siRNA in the End1/E6E7 cells and HeLa cells. β-actin: 520 bp, TKT: 176 bp, TKTL1: 150 bp, TKTL2: 146 bp.

The relative expression level of each member of the transketlase gene family was determined by real-time PCR in HeLa and End1/E6E7 cells. In the HeLa cells without transfection, the expression level of the TKTL1 gene was higher compared to the expression of TKT and TKTL2 gene. In the End1/E6E7 cells without transfection, the expression level of the TKTL1 gene was lower compared to the expression of TKT and TKTL2 gene. There was no significant difference in the expression level of TKT and TKTL2 gene between the HeLa and End1/E6E7 cells without transfection (*P *> 0.05). However, the expression level of TKTL1 gene was significantly down-regulated in the HeLa and End1/E6E7 cells transfected with siRNA TKTL1 construct compare with the cells transfected with control plasmid or cells without transfection (*P *< 0.01). There was no significant difference in the expression level of TKT and TKTL2 gene among the cells without transfection, transfected with control plasmid and transfected with siRNA in the HeLa or End1/E6E7 cell line (Fig 1, Table 1).

**Table 1:** The relative change in expression of transketolase gene family members in the human uterine cervix cancer and normal cervical epithelial cells

	**HeLa cells**	**HeLa cells**	**HeLa cells**	**End1/E6E7 cells**	**End1/E6E7 cells**
**Gene**	**(No transfection)**	**(control siRNA)**	**(siRNA)**	**(control siRNA)**	**(siRNA)**

TKT	1.05 ± 0.12	0.98 ± 0.09	1.06 ± 0.11	0.96 ± 0.10	1.02 ± 0.08

TKTL1	8.62 ± 0.92	8.43 ± 0.78	0.15 ± 0.02	1.03 ± 0.11	0.17 ± 0.03

TKTL 2	0.89 ± 0.10	1.12 ± 0.13	1.06 ± 0.11	0.99 ± 0.07	1.02 ± 0.09

The expression level of transketolase gene family members was analyzed using real-time quantitative PCR. The relative expression amount of target gene in the HeLa and End1/E6E7 cells was calculated using the 2 ^-ΔΔCT ^method. The relative expression amount of target gene in the HeLa cell was normalized to β-actin and relative to the expression of End1/E6E7 cells untreated. The relative expression amount of target gene in the End1/E6E7 cells (transfected with control plasmid and transfected with siRNA) was normalized to β-actin and relative to the expression of cells untreated.

### Transketolase activity in human uterine cervix cancer and normal cervical epithelial cells

**Figure 2:**
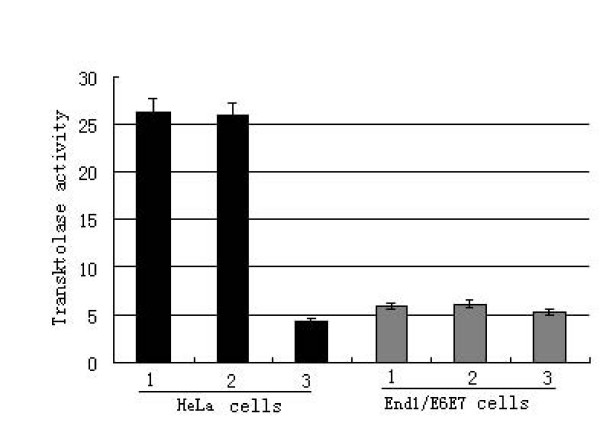
**The effect of anti-TKTL1 siRNA on transketolase activity in the HeLa cells and End1/E6E7 cells**. 1: the cells without transfection, 2: the cells transfected control plasmid, 3: the cells transfected siRNA. The total transketolase activity was significantly increased in the HeLa cells without transfection compared to that in the End1/E6E7 cells without transfection. The total transketolase activity was significantly decreased in the HeLa cells transfected siRNA. There were no significant difference existed in total transketolase activity after transfected siRNA in the End1/E6E7 cells.

In order to estimate whether TKTL1 plays an important role in the total transketolase activity in the uterine cervix cancer and normal cervical epithelial cells, the total transketolase activity was measured in the cells without transfection, transfected with control plasmid and transfected with siRNA. We found that no significant difference existed in total transketolase activity between HeLa cells transfected with control plasmid and without transfection. In contrast, the total transketolase activity was significantly decreased in the HeLa cells transfected siRNA. There were no significant difference existed in total transketolase activity among the End1/E6E7 cells without transfection, transfected with control plasmid and transfected with siRNA. The total transketolase activity was significantly increased in the HeLa cells without transfection compared to that in the End1/E6E7 cells without transfection. These results demonstrated that TKTL1 play a key role in the total transketolase activity in the HeLa cells, while it is not important in the total transketolase activity in End1/E6E7 cells (Fig 2).

### The effect of siRNA TKTL1 on cell cycle in HeLa and End1/E6E7 cell line

To estimate the effect of siRNA TKTL1 on cell cycle we transfected HeLa and End1/E6E7 cells using above different plasmids, respectively. Each test was repeated three times. In comparison to HeLa cells transfected with control plasmid, or cells without transfection, after transfection with siRNA TKTL1, the percentage of apoptotic cells and G_0_/G_1 _stage cells was increased, and the percentage of S stage cells showed no significant change, while the percentage of G2/M stage cells was significantly reduced. There was no significant difference existed in cell cycle among the End1/E6E7 cells without transfection, transfected with control plasmid and transfected with siRNA (Table 2).

**Table 2:** The effect of siRNA TKTL1 on cell cycle in the End1/E6E7 cells and HeLa cells (The number of cells, %)

	**No transfection**	**Control plasmid**	**siRNA**
End1/E6E7 cells	M1:3.26 ± 0.12	5.12 ± 0.18	5.32 ± 0.16

	M2:72.68 ± 3.52	71.96 ± 3.26	72.38 ± 3.45

	M3:11.32 ± 0.68	10.84 ± 0.62	11.24 ± 0.63

	M4:12.74 ± 0.72	12.08 ± 0.70	11.06 ± 0.66

HeLa cells	M1:4.07 ± 0.16	4.62 ± 0.23	5.57 ± 0.21

	M2:54.24 ± 2.36	55.36 ± 2.75	69.02 ± 2.98

	M3:15.71 ± 0.78	15.84 ± 0.81	15.93 ± 0.84

	M4:25.98 ± 1.24	24.18 ± 1.16	9.48 ± 0.56

M1: the percentage of apoptotic cells, M2: G_0_/G_1 _stage cells, M3: S stage cells, M4: G2/M stage cells. In the End1/E6E7 cells, there was no significant difference existed in cell cycle among the cells without transfection, transfected with control plasmid and transfected with siRNA. In the HeLa cells, after transfection with siRNA TKTL1, the percentage of G_0_/G_1 _stage cells was increased, the percentage of G2/M stage cells was significantly reduced.

### The effect of siRNA TKTL1 on cell proliferation in HeLa and End1/E6E7 cell line

**Figure 3:**
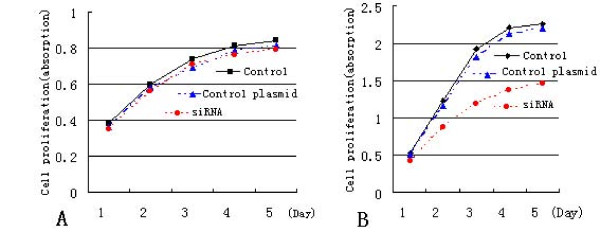
**The effect of anti-TKTL1 siRNA on proliferation of End1/E6E7 cells and HeLa cells**. In the End1/E6E7 cells (A), There was no significant difference of cell proliferation among the cells without transfection, transfected with control plasmid and transfected with siRNA. In the HeLa cells (B), cell proliferation was significantly inhibited after transfected siRNA TKTL1 construct.

To examine the effect of siRNA TKTL1 on cell proliferation, the absorption values of one culture plate from each group cells were detected by using MTT at 490 nm on daily basis for a period of five days. The growth curve of each cell group showed that cell proliferation was slower in the HeLa cells transfected siRNA TKTL1 construct than the cells transfected with control plasmid, or cells without transfection (Fig 3). There was no significant difference of cell proliferation among the End1/E6E7 cells without transfection, transfected with control plasmid and transfected with siRNA. Those results suggested that cells proliferation was inhibited by transfected siRNA TKTL1 construct in the HeLa cells. While, there was no significant difference on cell proliferation in normal cells after transfected siRNA TKTL1 construct.

## Discussion

Tumor cells need a large amount of energy and nucleic acids to survive and grow. For most of their energy needs, malignant cells typically depend on glycolysis mainly, the anaerobic breakdown of glucose into ATP [1]. Malignant cells characteristically exhibit an increased reliance on anaerobic metabolism of glucose to lactic acid even in the presence of abundant oxygen had been described by Warburg 80 years ago [2]. But, this theory was gradually discredited. Latter Following the development of bioenergetics, recent studies demonstrated that energy metabolism in malignant cells is significantly enhanced compared to those in the normal cells, especially glycometabolism [1]. The malignant cells maintain ATP production by increasing glucose flux because anaerobic metabolism of glucose to lactic acid is substantially less efficient than oxidation to CO_2 _and H_2_O. PET imaging has demonstrated a direct correlation between tumor aggressiveness and the rate of glucose consumption [10,11]. It is now widely applied to human cancers because the vast majority of primary and metastatic tumors demonstrate substantially increased glucose uptake compared with normal tissue. The majority of nucleic acids for tumor cells growth are generated directly or indirectly from the nonoxidative pathway of the PPP. Transketolase is a crucial enzyme in the nonoxidative pathway of the PPP. It has been presumed that transketolase activity possibly plays an important role in the tumor cell proliferation. Boros [4] found that the PPP was directly involved in degradation of glucose and played a crucial role in nucleic acid ribose synthesis utilising glucose carbons in tumor cells. Coy [9] indicated that tumor cells which upregulate transketolase enzyme reactions can use glucose as an energy source through nonoxidative generation of ATP. Using metabolic control analysis methods and oxythiamine, Comin-Anduix [12] demonstrated that transketolase enzyme reactions determine cell proliferation in the Ehrlich's ascites tumor model.

Ttransketolase gene family remember include transketolase(TKT), transketolase-like gene 1 (TKTL1) and transketolase-like gene 2 (TKTL2). The relative contributions of transketolase gene family to energy metabolism and proliferation of uterine cervix cancer cell have not been investigated. In the present study, the total transketolase activity was measured in the HeLa cells and End1/E6E7 cells. We found that the total transketolase activity was significantly increased in the HeLa cells compare to End1/E6E7 cells. In order to estimate whether TKTL1 play an important role in the total transketolase activity in the HeLa cells and End1/E6E7 cells, the relative expression level of each member of the transketlase gene family was determined by real-time PCR in HeLa and End1/E6E7 cells. We found that there was no significant difference in the expression level of TKT and TKTL2 gene between the HeLa and End1/E6E7 cells, the expression level of the TKTL1 gene was high in the HeLa cells compared to End1/E6E7 cells. After transfected siRNA TKTL1 construct, the total transketolase activity was significantly decreased in the HeLa cells. However, there was no significant difference existed in total transketolase activity in the End1/E6E7 cells after transfected siRNA TKTL1 construct. These results demonstrated that TKTL1 play a key role in the total transketolase activity in the HeLa cells, yet not so in the End1/E6E7 cells. In order to explore the effect of TKTL1 on cell proliferation of cervix cancer cell, we transfected the HeLa cells and End1/E6E7 cells with siRNA TKTL1 construct. Our results demonstrated that the proliferation of HeLa cells was significantly inhibited, and the cells were blocked in G_0_/G_1 _stage. Whereas, there was no significant change in cell proliferation and cell cycle in the End1/E6E7 cells. So, we think that strong TKTL1 expression was correlated to fast proliferation of cervix cancer cells. Lanbein [5] found that strong TKTL1 protein expression was correlated to invasive colon and urothelial tumours and to poor patient outcome. Staiger [6] demonstrated that TKTL1 upregulation is a common phenomenon in gastric cancer and cancer of the gastroesophageal junction. Földi indicated that TKTL1 expression in 86% of breast cancer specimens with 45% showing high expression levels. Langbein[13] demonstrated that Transketolase was more elevated in metastasizing renal cell cancer and TKTL1 protein was significantly overexpressed in progressing renal cell cancer. Our previous study revealed that TKTL1 play an important role in cell proliferation of colon cancer, hepatoma and nasopharyngeal carcinoma [14-16]. These results indicated that TKTL1 could be seen as a potential target for novel anti-transketolase cancer therapies.

In a word, TKTL1 plays an important role in total transketolase activity and proliferation of tumor cells in uterine cervix cancer. After the expression of TKTL1 was silenced, the proliferation of uterine cervix cancer cells was significantly inhibited; there was no significant change in normal cervical epithelial cells. We think that the most effective way to inhibit tumor proliferation should be to block the generation of energy or nucleic acids for tumor growth. So, we believe TKTL1 gene might become a novel hot spot of study in anticancer therapy.

## Competing interests

The authors declare that they have no competing interests.

## Authors' contributions

HC carried out the cell proliferation assay and drafted the manuscript. JXY participated in the design of the study and performed the statistical analysis. SHY carried out cell culture and plasmid construction. HD carried out transfection and RT-PCR. RWZ carried out measurements of transketolase activity. SZ conceived of the study, and participated in its design and coordination. All authors read and approved the final manuscript.
